# Aggressive re-warming at 38.5 °C following deep hypothermia at 21 °C increases neutrophil membrane bound elastase activity and pro-inflammatory factor release

**DOI:** 10.1186/s40064-016-2084-x

**Published:** 2016-04-21

**Authors:** Min Tang, Xiao-gang Zhao, Yi He, John Yan Gu, Ju Mei

**Affiliations:** Department of Cardiothoracic Surgery, Shanghai Xinhua Hospital Affiliated to Shanghai Jiaotong University School of Medicine, Shanghai, China; Department of Thoracic Surgery, Shanghai Pulmonary Hospital Affiliated to Tongji University, Shanghai, China; Department of Biomedical Engineering, University Medical Centre Groningen, Groningen, The Netherlands

**Keywords:** Hyperthermia, Hypothermia, Inflammation, Interleukin, Membrane bound elastase, Neutrophil

## Abstract

**Background:**

Cardiopulmonary bypass (CPB) is often performed under hypothermic condition. The effects of hypothermia and re-warming on neutrophil activity are unclear. This study aimed to compare the effects of different hypothermia and re-warming regimens on neutrophil membrane bound elastase (MBE) activity and the release of pro-inflammatory factors from neutrophils.

**Methods:**

Human neutrophils were exposed to different hypothermia and re-warming regimens. MBE activity and the release of interleukin (IL)-β1, IL-6, IL-8, and tumor necrosis factor (TNF)-α were measured.

**Results:**

Neutrophil MBE activity was significantly reduced after 60-min moderate (28 °C) or deep (21 °C) hypothermic treatment. Compared with normothermic (37 °C) re-warming, aggressive re-warming (38.5°) for 120 min following deep hypothermia (21 °C) dramatically increased neutrophil MBE activity (*P* < 0.05). Co-incubation of neutrophils with platelet-rich plasma further increased MBE activity significantly under all the tested temperature regimens. IL-β1 release from neutrophils was significantly higher after deep hypothermia (21 °C) followed by normothermic (37 °C) re-warming than after moderate hypothermia (28 °C) followed by normothermic re-warming (*P* < 0.05). Aggressive re-warming (38.5°) following deep hypothermia significantly increased the release of IL-β1, IL-8, and TNF-α from neutrophil compared with moderate re-warming (37 °C) (all *P* < 0.05).

**Conclusion:**

Aggressive re-warming following deep hypothermia may contribute to CPB-associated tissue injury by increasing neutrophil MBE activity and stimulating pro-inflammatory factor release, thus, should be avoided. The optimal hypothermic temperature of CPB should be determined based on patient clinical characteristics and surgery type.

## Background

Cardiopulmonary bypass (CPB), a common procedure used in cardiac and thoracic surgery, is usually performed under hypothermia to reduce the body’s metabolic rate and decrease oxygen consumption so to protect organs, such as the brain, kidneys, lungs, and heart from ischemic injuries. Despite the hypothermic approach, CPB still cause adverse systemic inflammatory responses, consequently leading to postoperative morbidity and mortality (Liguori et al. 2014). In addition to those well-known reasons causing CPB-induced adverse systemic inflammation, such as the contact of blood with the artificial surface of a CPB circuit and ischemia–reperfusion during CPB, the re-warming process during re-perfusion after CPB might also be a contributing factor.

Currently, several hypothermia and re-warming regimens have been used in CPB (Grigore et al. 2009). Deep hypothermic circulatory arrest is often practiced for complex cardiothoracic surgeries, such as aortic surgery and repair of complex congenital defects, to create a bloodless surgical field and protect brain function (Lima et al. 2011). It has been shown that hypothermia during CPB exerts significant brain protection, suppresses the release of free radicals, and inhibits destructive enzymatic reactions (Hicks et al. 2000; Yager and Asselin 1996; Bernard et al. 2002; Safar and Kochanek 2002; Safar 2003). Different re-warming temperatures have also been tested. In addition to normothermic re-warming at 37 °C, aggressive re-warming at a higher temperature such as 38.5 °C has also been considered because it can considerably reduce the duration of CPB (Hata et al. 2009). However, Grigore et al. (2001, 2002) found that aggressive re-warming was significantly associated with poor neurocognitive outcomes. In addition, hyperthermic perfusion is discouraged in the current American guidelines for CPB management because of the risk of neurologic complications (Engelman et al. 2015). Although the impact of temperature regimens of CPB on neurologic function has been extensively studied, the effects of hypothermia and re-warming regimen on inflammatory responses remain unknown.

Activation of neutrophils is considered as one of the key mechanisms underlying CPB-induced inflammation (Butler et al. 1993). During CPB, neutrophils can be stimulated to secrete lactoferrin and elastase (Colman 1990), and the stimulated neutrophils have been found to release pro-inflammatory factor IL-8 (Altstaedt et al. 1996), leading to adverse systemic inflammation. The purpose of this study was to investigate the effects of different hypothermia and re-warming regimens on neutrophil activation in vitro by measuring the activity of neutrophil membrane bound elastase (MBE), which is considered as an inflammation indicator. The effects of different temperature regimens on pro-inflammatory factor release from neutrophils were also studied.

## Methods

### Blood sample collection

The study protocol was approved by the Institutional Review Board of Shanghai Xinhua hospital. Signed informed consent was obtained from each blood donor. Blood samples were collected from 15 healthy men at Shanghai Xinhua Hospital. A total of 20 mL whole blood was withdrawn from each donor and mixed immediately with 0.3 mL sterilized anticoagulant solution containing 10 % (w/v) ethylene diamine tetraacetic acid in phosphate-buffered saline (PBS). To prevent bacterial contamination, all the laboratory supplies used in the study were sterilized.

### Neutrophil isolation

Neutrophil isolation was performed according to the previous description (Hjorth et al. 1981). Briefly, the freshly collected whole blood samples were centrifuged at 160×*g* for 10 min at 4 °C. The plasma layer was then collected and diluted with PBS at 1:1 (v/v) ratio. A buffer for discontinuous gradient centrifugation was prepared by overlaying 4 mL of 55 % (v/v) Percoll (Sigma^®^, St. Louis, MI, USA) in 0.9 % (w/v) NaCl solution (ρ = 1.1026 g/mL) on top of 4 mL of 74 % Percoll in 0.9 % NaCl solution (ρ = 1.0776 g/mL). Four mL of plasma concentrate was subsequently overlaid on top of the gradient buffer and centrifuged at 350×*g* for 20 min at 4 °C. The neutrophil band, which was the layer between the 2 layers of Percoll, was carefully removed using a Pasteur pipette. The collected neutrophils were washed with PBS and centrifuged at 350×*g* for 15 min at 4 °C. The contaminated red blood cells in the neutrophil collection were removed by incubating the neutrophil with hypotonic lysis buffer (0.83 % w/v, NH_4_Cl, 0.0037 % w/v, Na_2_EDTA, 0.1 % w/v KHCO_3_) for 5 min at room temperature. Neutrophils were collected and washed by centrifugation at 160×*g* for 5 min. The purity and viability, which was determined by Giemsa stain and trypan blue (0.01 % w/v) exclusion, respectively, were all >95 %. Neutrophils were counted using a haemocytometer under a microscope, and diluted in PBS to a final concentration of 4 × 10^6^ cells/mL for future experiments.

### Platelet-rich plasma (PRP) preparation

Ten mL of plasma obtained by centrifugation of whole blood at 160×*g* for 10 min at 4 °C was further centrifuged at 350×*g* for 10 min at 4 °C. After centrifugation, 1 mL solution was removed from the bottom of the tube, which was considered as PRP. The 1 mL PRP was mixed with 20 μL sterile water containing 300 IU thrombin and incubated at 37 °C for 10 min. The final concentration of thrombin in PRP was 0.25 IU/mL.

### Hypothermic and re-warming treatment for neutrophils

**Fig. 1:**
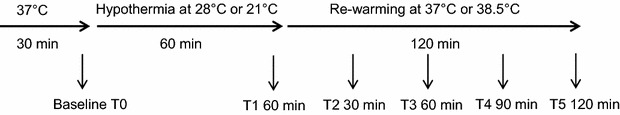
Protocol of hypothermia and re-warming and time point of MBE measurement

The protocol of hypothermia and re-warming is illustrated in Fig. 1. Neutrophils were aliquoted and treated with 4 different temperature regimens, respectively. For moderate hypothermia followed by normothermic re-warming treatment (28–37 °C), neutrophils were incubated at 37 °C for 30 min, 28 °C for 60 min, and then 37 °C for 120 min. In deep hypothermia followed by normothermic re-warming treatment (21–37 °C), neutrophils were incubated at 37 °C for 30 min, 21 °C for 60 min, and then 37 °C for 120 min. For deep hypothermia followed by aggressive re-warming treatment (21–38.5 °C), neutrophils were incubated at 37 °C for 30 min, 21 °C for 60 min, and then 38.5 °C for 120 min. In the control treatment, neutrophils were incubated at 37 °C for 210 min. Neutrophils were incubated with either PBS or PRP in each temperature treatment. Thermocycler (Gene Amp^®^ 2400, Applied Biosystem, USA) for polymerase chain reaction was used for the hypothermia and re-warming treatments according to our previous description (Tang et al. 2004, 2010). Aliquots of 50 μL neutrophil suspension either in PBS or mixed with PRP was prepared. The aliquots were treated with the four specific hypothermia and re-warming regimens, respectively. The thermocycler was pre-heated to 37 °C. The hypothermia and re-warming protocols were programmed before neutrophil samples were placed into the thermocycler. At each specific time point for MBE measurement, the corresponding neutrophil aliquots was quickly removed from the thermocycler and measured for MBE activity immediately. Three aliquots were used for each measurement.

### Determination of membrane-bound elastase (MBE) activity

MBE activity was measured at baseline (T0) and the following specific time points: T1: at the end of the hypothermia, T2: 30 min after re-warming, T3: 60 min after re-warming, T4: 90 min after re-warming, and T5: 120 min after re-warming. When neutrophils were incubated with PRP, MBE activity was only measured at 120-min re-warming (Fig. 1). MBE activity was measured according to the method described previously (Owen et al. 1995). Briefly, after temperature treatments, neutrophils were collected by centrifugation at 160×*g* at 25 °C for 5 min, fixed by paraformaldehyde (3 % w/v in PBS) and glutaraldehyde (1 % v/v in PBS, both from Sigma^®^, St. Louis, MI, USA) at 4 °C for 3 min, and then washed twice in PBS by centrifugation at 160×*g* at 4 °C for 15 min. The neutrophils were then re-suspended in PBS and MBE activity was analyzed by a substrate method. A 50 μL aliquot of neutrophil suspension was mixed with 150 μL of substrate solution containing 1 mM *N*-succinyl-(l-alanine)_3_-*p*-nitroanilide (Sigma^®^, St. Louis, MI, USA) dissolved in 200 mM Tris–HCl (pH 8.0), and the mixture was incubated at 37 °C for 60 min. The reaction was terminated by adding 5 μL of glacial acetic acid. Absorbance at 405 nm was recorded in a Bio-Rad plate reader (Bio-Rad^®^, Hercules, USA). A standard curve of elastase was prepared using different concentrations of standard elastase solution (Sigma^®^, St. Louis, MI, USA) in the substrate solution. MBE activity was expressed as ng enzyme/10^6^ neutrophils. The sensitivity of this assay was 0.5 ng enzyme/10^6^ neutrophils.

### ELISA analysis of pro-inflammatory factors

Neutrophils from each volunteer were treated with the 4 temperature regimens, respectively. At the end of each treatment, IL-β1, IL-6, IL-8, and TNF-α in the culture supernatants were analyzed by ELISA using a kit according to the protocol provided by the manufacturer (R&D System, USA).

### Statistical analysis

The statistical analysis was performed using the software SPSS 19.0. Data are presented as mean ± standard deviation. One-way ANOVA was used to compare MBE activity and inflammatory factor levels among different groups. *P* value was 2-sided and *P* < 0.05 was considered significantly different.

## Results

### Deep hypothermia followed by aggressive re-warming increased neutrophil MBE activity the most

**Fig. 2:**
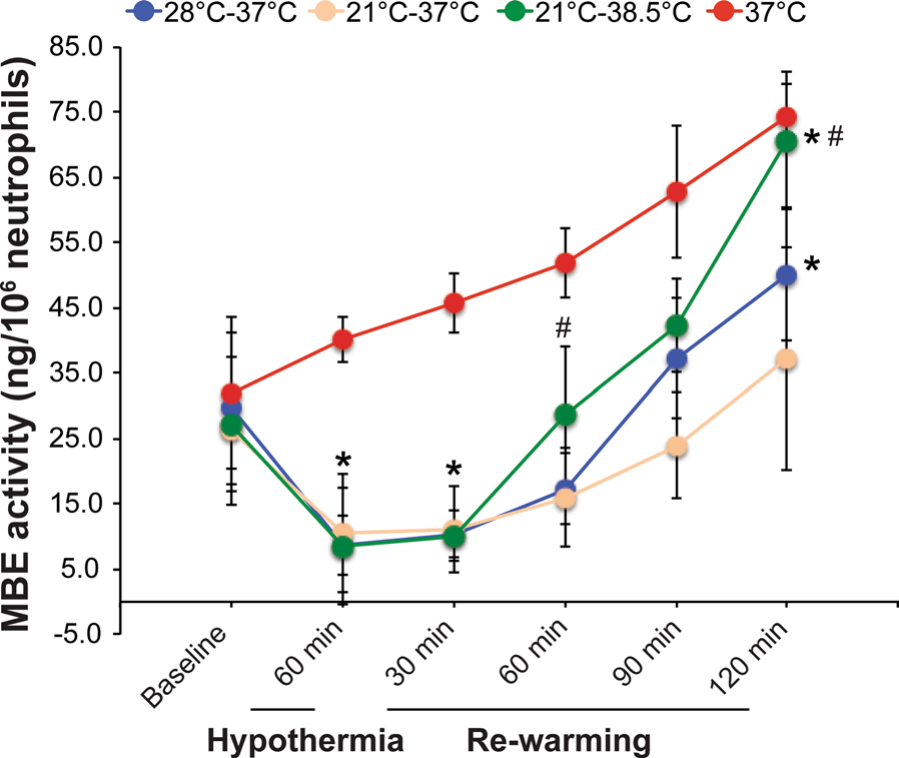
Neutrophil MBE activity at different time point of temperature regimens. Neutrophils were treated with the four temperature regimens, respectively. MBE activity was then measure at the specific time point. Average MBE activity was calculated, n = 15. Values were compared using one-way ANOVA. *Significant difference between the indicated values and the baseline values of the respective temperature regimen. ^#^Significant difference between aggressive re-warming (38.5 °C) and normothermic re-warming (37 °C). *P* < 0.05

**Fig. 3:**
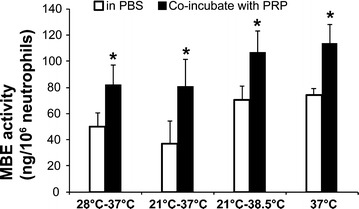
Co-incubation with PRP further increased neutrophil MBE activity. Neutrophils were co-incubated with PRP during different temperature treatments. MBE activity was measure at 120-min re-warming. MBE activity was compared between the co-incubation of neutrophils with PRP and the neutrophils alone. Comparison was analyzed by Student’s *t* test. *Significant difference between co-incubation with PRP and neutrophil alone, *P* < 0.05

MBE activity was reduced significantly after 60-min hypothermia (*P* < 0.05) compared with the baseline values, and the reduction was similar after moderate (28 °C) or deep (21 °C) hypothermia (8.6 ± 4.3 ng/10^6^ cells vs. 10.5 ± 9.1 ng/10^6^, *P* = 0.837, Fig. 2). MBE activity remained at significant low levels after 30-min re-warming at 37 °C or 38.5 °C. At 60-min re-warming, MBE activity was significantly higher in neutrophils treated with aggressive re-warming (38.5 °C) than in neutrophils treated with normothermic re-warming (37 °C) (*P* < 0.05). At 120-min re-warming, MBE activity of neutrophils treated with deep hypothermia followed by normothermic re-warming (21–37 °C) was at the similar level to the baseline value (35.2 ± 17.0 ng/10^6^ cells vs. 26.2 ± 11.4 ng/10^6^ cells, *P* > 0.05). In contrast, both deep hypothermia followed by aggressive re-warming (21–38.5 °C) and moderate hypothermia followed by normothermic re-warming (28–37 °C) increased MBE activity significantly compared with the baseline values (70.6 ± 10.5 ng/10^6^ cells vs. 27.2 ± 10.2 ng/10^6^ cells and 50.2 ± 10.1 ng/10^6^ cells vs. 29.7 ± 11.6 ng/10^6^ cells, respectively, all *P* < 0.05, Fig. 2) at the end of 120-min re-warming. MBE activity under the control treatment increased continuously through the experiment, and reached the highest 2 h after incubation at 37 °C (74.3 ± 5.1 ng/10^6^), which was similar to the MBE activity 2 h after aggressive re-warming (70.6 ± 10.5 ng/10^6^, *P* > 0.05). Co-incubation of neutrophils with PRP during the temperature treatments significantly increased MBE activity compared with neutrophil alone for all the temperature regimens (all *P* < 0.05, Fig. 3).

### Aggressive re-warming increased the release of pro-inflammatory factors from neutrophils

**Fig. 4:**
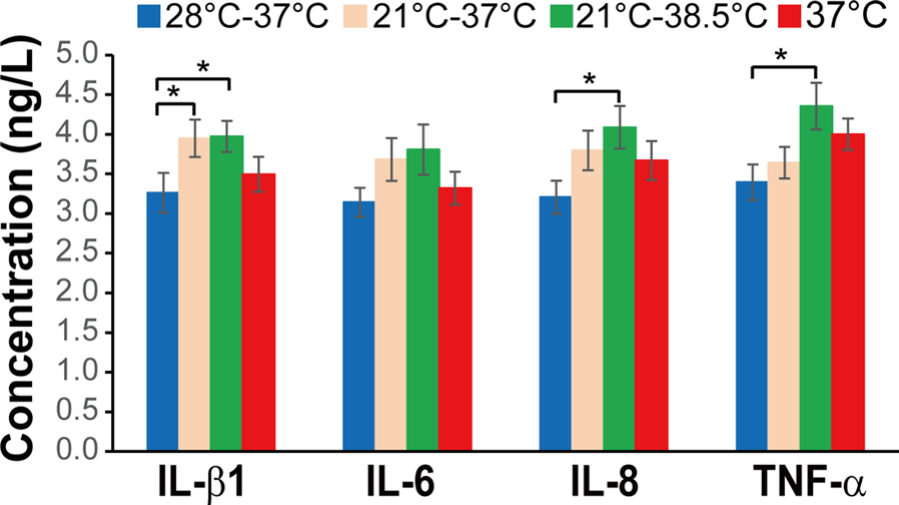
Aggressive re-warming followed by deep hypothermia significantly increased the release of pro-inflammatory factors. Neutrophils were treated with different temperature regimens. The concentrations of IL-β1, IL-6, IL-8, and TNF-α in the incubation media were measured at 120-min re-warming by ELISA. Comparison was analyzed by one-way ANOVA. *Significant difference between the indicated groups, *P* < 0.05

ELISA assay on pro-inflammatory factor secretion showed that deep hypothermia followed by aggressive re-warming regimen (21–38.5 °C) significantly increased the release of IL-β1, IL-8, and TNF-α from neutrophils compared with moderate hypothermia followed by normothermic re-warming regimen (28–37 °C) (All *P* < 0.05, Fig. 4). Deep hypothermia followed by normothermic re-warming regimen (21–37 °C) increased IL-β1 release from neutrophils significantly compared with moderate hypothermia followed by normothermic re-warming (28–37 °C) (3.95 ± 0.236 ng/L vs. 3.26 ± 0.253 ng/L, *P* < 0.05, Fig. 4).

## Discussion

In this current study, both moderate (28 °C) and deep hypothermia (21 °C) significantly decreased neutrophil MBE activity in vitro. Interestingly, deep hypothermia followed by normothermic re-warming (21°–37 °C) kept neutrophil MBE activity at a lower or similar level to the baseline value. In contrast, moderate hypothermia followed by normothermic re-warming (28°–37 °C) resulted in a fast increase in neutrophil MBE activity after 60-min re-warming, and the MBE activity at the end of 2 h of re-warming was about 1.7 folds of the baseline value. In vivo studies showed similar results. Significantly lower elastase activity has also been observed in the blood samples of patients undergoing cold (26.3 ± 0.5 °C) CPB compared with warm (31.8 ± 0.4 °C) CPB (Menasché et al. 1995). Elevation of neutrophil elastase is associated with CPB-induced tissue damage and inflammation (Kawabata et al. 2002; Shapiro 2002). Thus, these results suggest that CPB at a low temperature may reduce CPB-associated tissue damage and inflammation by keeping neutrophil elastase activity at low levels. A continuous increase in neutrophil MBE activity in the control treatment (continuous 37 °C) suggests that neutrophils may be constantly activated at 37 °C, further supporting that hypothermic treatment during CPB may reduce CPB-associated organ damage. On the other hand, this current study also showed that hypothermia at low temperature (21 °C) significantly increased the release of the pro-inflammatory factor IL-β1 from neutrophils compared with moderate hypothermic temperature (28 °C). The effects of hypothermia on neutrophil elastase activity and pro-inflammatory factor release appear to be opposite. Thus, the optimal hypothermic temperature of CPB may not be the lower the better and should be determined based on patient clinical characteristics. Clinical studies also showed complex effects of hypothermic CPB on patient outcome.

Lima et al. (2011) retrospectively reviewed 245 patients undergoing proximal arch replacement with deep hypothermic circulation arrest at 18.0 ± 2.1 °C and found that both neurologic and non-neurologic outcomes were excellent in these patients. The results of this current in vitro study indicate that the beneficial effects of deep hypothermia could be mediated by inhibition of neutrophil MBE activity. However, deep hypothermic CPB might have potential adverse consequences, such as an increased risk of postoperative bleeding (Harrington et al. 2004). In addition, deep hypothermia will also increase the duration of CPB, which consequently will deteriorate CPB-induced inflammation and tissue injury. Reich et al. (1999) reported that in senior patients, deep hypothermia was associated with memory and find motor deficits and prolonged hospital stay. Thus, the optimal hypothermic temperature during CPB should be determined based on the type of surgery and the clinical characteristics of patients.

This current study showed that during the first 30 min of the re-warming, neutrophil MBE activity remained at low levels and was similar in the normothermic and the hyperthermic re-warming groups. This result indicates that neutrophils may need a minimum of 30 min to recover from the 2-h hypothermic treatment although the temperature of the neutrophils may reach the re-warming temperature rapidly. After the recovery period, the response of neutrophils to normothermia and hyperthermia was different. Hyperthermic re-warming increased neutrophil MBE activity at higher extent than normothermic re-warming. MBE activity was increased quickly and dramatically during aggressive hyperthermic re-warming (38.5 °C) and reached approximately threefolds of the baseline value after 2 h of re-warming at 38.5 °C. These findings suggest that fast re-warming during CPB may cause excessive neutrophil activation and exacerbate systemic inflammation.

Additionally, this current study also demonstrated that aggressive re-warming following deep hypothermia (21–38.5 °C) significantly increased the release of pro-inflammatory factors, IL-β1, IL-8, and TNF-α compared with the moderate temperature treatment (28–37 °C). TNF-α has been well recognized to contribute to CPB-induced systemic inflammation and tissue damage (Hill 1998). These findings suggest that aggressive re-warming following deep hypothermia in CPB not only can dramatically increase neutrophil elastase activity but also can stimulate the release of pro-inflammatory factors, consequently leading to tissue damage and systemic inflammation. Clinical and preclinical studies also demonstrated that adverse effects were associated with fast re-warming. Newman et al. (1995) found that fast re-warming contribute significantly to the decline in complex spatial and figure memory of elderly patients undergoing cardiac surgery. Grigore et al. (2001, 2002) also showed that fast re-warming was significantly associated with poor postoperative neurocognitive outcomes . In addition, in a swine model of lethal hemorrhage, Alam et al. (2006) demonstrated that fast re-warming from deep hypothermia reduced the animal survival substantially compared with medium rate of re-warming. Thus, aggressive rewarming following deep hypothermia in CPB should be avoided.

Compared with the other temperature treatment, neutrophil MBE activity was the highest at the end of the continuous 37 °C treatment, whereas the pro-inflammatory factor release at 37 °C treatment was similar to that in the normothermic re-warming group. These results suggest that neutrophil MBE activity may be elevated under continuous 37 °C treatment but pro-inflammatory factor release may not. Thus, different molecular and cellular mechanisms appear to be involved in the stimulation of MBE activity and the induction of pro-inflammatory factor release in neutrophils.

In addition to re-warming, other pro-inflammatory factors can further activate neutrophils. In this current study, we found that co-incubation with PRP significantly further increased MBE activity in all temperature regimens. These results indicated that anti-inflammation therapies in addition to hypothermia, such as steroids, are required to effectively prevent or reduce CPB-induced inflammatory responses. The neutrophil elastase inhibitor, sivelestat sodium hydrate, has been shown to reduce the serum levels of pro-inflammatory cytokines IL-6 and IL-8 and attenuate pulmonary dysfunction in patients undergoing CPB (Fujii et al. 2010; Kohira et al. 2013).

In this current in vitro model, although the temperature change of neutrophils during hypothermia and re-warming is much faster than that of the human body exposed to CPB, this in vitro model mimics the temperature change of blood in a CPB machine. In a CPB machine, the re-warming process is started after the water tank of the CPB machine reaches the programmed re-warming temperature, and during the phase of re-warming, the hypothermic blood in the CPB tube encounter the re-warming temperature. This in vitro model may be used to optimize the hypothermic and re-warming temperature regimen in CPB.

## Conclusion

The findings of this current study suggest that aggressive re-warming following deep hypothermia may contribute to CPB-induced tissue injury and systemic inflammation by increasing neutrophil MBE activity and stimulating pro-inflammatory factor release. Thus, aggressive re-warming in CPB should be avoided and the optimal hypothermic temperature of CPB should be determined based on patient clinical characteristics and surgery type.
